# Analysis of individual alpha frequency in a large cohort from a tertiary memory center

**DOI:** 10.1111/ene.16424

**Published:** 2024-08-01

**Authors:** Giordano Cecchetti, Federica Agosta, Elisa Canu, Silvia Basaia, Giulia Rugarli, Davide G. Curti, Federico Coraglia, Marco Cursi, Edoardo G. Spinelli, Roberto Santangelo, Francesca Caso, Giovanna Franca Fanelli, Giuseppe Magnani, Massimo Filippi

**Affiliations:** ^1^ Neurology Unit IRCCS San Raffaele Scientific Institute Milan Italy; ^2^ Neurophysiology Service IRCCS San Raffaele Scientific Institute Milan Italy; ^3^ Neuroimaging Research Unit, Division of Neuroscience IRCCS San Raffaele Scientific Institute Milan Italy; ^4^ Vita‐Salute San Raffaele University Milan Italy; ^5^ Neurorehabilitation Unit IRCCS San Raffaele Scientific Institute Milan Italy

**Keywords:** Alzheimer disease, biomarkers, differential diagnosis, EEG, individual alpha frequency

## Abstract

**Background and Purpose:**

Precise and timely diagnosis is crucial for the optimal use of emerging disease‐modifying treatments for Alzheimer disease (AD). Electroencephalography (EEG), which is noninvasive and cost‐effective, can capture neural abnormalities linked to various dementias. This study explores the use of individual alpha frequency (IAF) derived from EEG as a diagnostic and prognostic tool in cognitively impaired patients.

**Methods:**

This retrospective study included 375 patients from the tertiary Memory Clinic of IRCCS San Raffaele Hospital, Milan, Italy. Participants underwent clinical and neuropsychological assessments, brain imaging, cerebrospinal fluid biomarker analysis, and resting‐state EEG. Patients were categorized by amyloid status, the AT(N) classification system, clinical diagnosis, and mild cognitive impairment (MCI) progression to AD dementia. IAF was calculated and compared among study groups. Receiver operating characteristic (ROC) analysis was used to calculate its discriminative performance.

**Results:**

IAF was higher in amyloid‐negative subjects and varied significantly across AT(N) groups. ROC analysis confirmed IAF's ability to distinguish A–T–N– from the A+T+N+ and A+T–N+ groups. IAF was lower in AD and Lewy body dementia patients compared to MCI and other dementia types, with moderate discriminatory capability. Among A+ MCI patients, IAF was significantly lower in those who converted to AD within 2 years compared to stable MCI patients and predicted time to conversion (*p* < 0.001, *R* = 0.38).

**Conclusions:**

IAF is a valuable tool for dementia diagnosis and prognosis, correlating with amyloid status and neurodegeneration. It effectively predicts MCI progression to AD, supporting its use in early, targeted interventions in the context of disease‐modifying treatments.

## INTRODUCTION

The emergence of disease‐modifying treatments for Alzheimer disease (AD) necessitates precise and timely diagnostic capabilities for optimal therapeutic interventions. However, the financial constraints, invasiveness, and restricted availability of advanced neuroimaging techniques and lumbar puncture for cerebrospinal fluid (CSF) AD biomarkers pose substantial obstacles to broad diagnostic coverage.

Electroencephalography (EEG) may offer a promising solution. Characterized by a noninvasive and cost‐effective nature, EEG may detect neural aberrations associated with various dementia subtypes [1, 2]. The individual alpha frequency (IAF), representing the dominant frequency in the background posterior cerebral activity, is easily obtainable from clinical EEG recordings and has demonstrated significant differentiation among dementia forms [2, 3, 4].

In this cross‐sectional and longitudinal retrospective study, we sought to fill a crucial gap in the literature by exploring IAF associations with CSF AD biomarkers, neuroimaging markers, and the risk of progression to dementia in a sizable and systematically characterized population of cognitively impaired patients from a tertiary memory center.

## METHODS

A total of 907 patients with cognitive impairment who underwent lumbar puncture for CSF AD biomarkers measurement as part of the standard clinical pathway were screened for inclusion from those evaluated at the Memory Clinic, Neurology Unit of IRCCS San Raffaele Hospital between January 2017 and December 2022. The inclusion criteria comprised a clinical diagnosis of mild cognitive impairment (MCI) or dementia, brain imaging scan (structural or functional), and resting‐state 19‐channel EEG, acquired as part of the diagnostic pathway. Excluded were patients with significant cognitive decline due to brain lesions, other clinically relevant systemic/neurological/major psychiatric disorders, drug/alcohol abuse, and evidence of epileptiform activity on EEG.

Following the AT(N) framework [5], the final sample was stratified by amyloid status (A+, A–, based on CSF Aβ42 or Aβ42/Aβ40 ratio) alone and by the entire AT(N) profile, involving amyloid status, tau pathology (T+, T–, based on CSF phosphorylated tau [pTau]), and neurodegeneration (N+, N–, based on CSF total tau, presence of atrophy according to the global cortical atrophy scale [6] at structural neuroimaging [Atrophy+ if the score was ≥2 in at least one brain region on either magnetic resonance imaging [MRI] or computed tomography, otherwise Atrophy–], and/or hypometabolism on fluorodeoxyglucose positron emission tomography [FDG‐PET; PET+, PET–]).

Patients were further categorized based on their clinical diagnosis at hospital discharge, including amnestic MCI (aMCI) [7], nonamnestic MCI, multidomain MCI, typical AD, early onset dementia due to AD [5, 8], behavioral variant frontotemporal dementia (bvFTD) [9], Lewy body dementia (LBD) [10], mixed AD, and vascular dementia [11].

Clinical data on A+ MCI patients' progression to full AD dementia were also collected. These patients were grouped into converters (A+ MCIc), who transitioned within 2 years, and stable MCI (A+ MCIs), who did not convert in 2 years.

EEGs were acquired in resting awake condition on a computer‐based system using 19 standard 10/20 electrode locations with linked ear reference [12]. EEG traces were visually inspected, and segments containing artifacts were rejected. EEG spectral analysis involved averaging the fast Fourier transform of at least 100 2‐s nonoverlapping epochs, tapered by Hanning window, under closed‐eye conditions. Power spectra of C3, C4, P3, P4, O1, and O2 were averaged to obtain a single mean power spectrum. Absolute power values were normalized into relative power. IAF [2], corresponding to the peak in the mean power spectrum within the extended alpha range (7–13 Hz) [2], was calculated using custom MATLAB (v9.10.0‐R2021a, MathWorks, Natick, MA, USA) routines. To validate the peak in the EEG spectrum representing IAF, we assessed its shape and quality. Metrics like first derivative, kurtosis index, and slope of best fit lines were used in the analysis. If the IAF was near the range limits (7–13 Hz), the analysis was extended by 3 Hz beyond this range, and the raw EEG traces were inspected to ensure that the posterior rhythms matched the frequencies of the spectral peaks.

Analyses were computed using R software, and the level of significance was set at *p* < 0.05. Clinical–demographic and cognitive variables were compared among study groups using Fisher exact test, analysis of variance, or age‐, sex‐, and education‐adjusted analysis of covariance (ANCOVA) models. Pearson coefficient was used to assess correlations between IAF and age, disease duration, Mini‐Mental State Examination (MMSE), and CSF pTau/Aβ42 ratio for the entire sample. IAF values were compared among AT(N) groups and clinical diagnostic groups and between A+ MCIc and A+ MCIs, utilizing age‐/sex‐/education‐adjusted and Bonferroni‐corrected ANCOVA models. Receiver operating characteristic (ROC) analysis evaluated the discriminative accuracy of IAF in selected pairwise comparisons. A linear regression model was implemented to investigate whether baseline IAF in A+ MCI patients predicted time of conversion to full‐blown AD dementia, adjusting for age, sex, and education.

## RESULTS

After the revision of inclusion and exclusion criteria, 375 patients were included in this study (Table 1). All EEGs were acquired before the initiation of dementia‐specific treatments (e.g., anticholinesterase inhibitors, memantine). Pearson correlation tests showed negligible to small correlation coefficients between IAF and demographic features, pTau/Aβ42 ratio, and global cognition (age: *r* = −0.06, *p* = 0.19; disease duration: *r* = 0.04, *p* = 0.39; pTau/Aβ42 ratio: *r* = −0.13, *p* = 0.01; MMSE: *r* = 0.29, *p* < 0.01).

**TABLE 1:** Demographic and clinical features of patients stratified according to amyloid status, AT(N) system, and clinical syndrome.

	*n*	Demographic features				CSF biomarkers		Clinical features		EEG features
		Age, years	Sex, F|M, % F	Education, years	Disease duration, years	Tau/Aβ42 ratio	pTau/Aβ42 ratio	MMSE	CDR	IAF
A+	227	71.10 ± 7.81	128|99 (56.39%)	10.60 ± 4.38	2.86 ± 2.19	1.36 ± 0.90	0.23 ± 0.17	21.60 ± 5.33	0.87 ± 0.71	8.34 ± 1.51
A−	148	71.40 ± 7.84	79|69 (53.38%)	9.78 ± 4.27	3.03 ± 2.14	0.52 ± 0.38^a^	0.08 ± 0.05^a^	23.40 ± 5.10^a^	0.74 ± 0.62	8.87 ± 1.45^a^
A−T−N−	18	68.90 ± 10.20	13|5 (72.22%)	10.00 ± 3.22	3.19 ± 2.25	0.31 ± 0.10^b^	0.05 ± 0.02^b^	25.60 ± 3.86^b^	0.50 ± 0.31	9.72 ± 0.99^b^, ^c^
A+T+N+	141	71.20 ± 7.74	84|57 (59.57%)	10.40 ± 4.46	2.90 ± 2.20	1.73 ± 0.90	0.30 ± 0.17	20.30 ± 5.53	0.96 ± 0.74	8.27 ± 1.46
A+T−N+	73	71.10 ± 8.00	34|39 (46.57%)	10.70 ± 4.23	2.78 ± 2.23	0.70 ± 0.36^b^	0.10 ± 0.10^b^	23.50 ± 4.21^b^	0.75 ± 0.64	8.32 ± 1.57
A−T−N+	71	71.10 ± 7.99	28|43 (39.43%)	9.46 ± 4.62	2.60 ± 2.02	0.31 ± 0.20^b^, ^c^	0.02 ± 0.05^b^, ^c^	23.30 ± 5.02^b^	0.75 ± 0.52	8.82 ± 1.29
A−T+N+	56	72.40 ± 6.76	35|21 (62.50%)	10.40 ± 4.10	3.48 ± 2.23	0.83 ± 0.40^b^, ^d^, ^e^	0.13 ± 0.05^b^, ^e^	22.90 ± 5.45^b^	0.81 ± 0.72	8.82 ± 1.66
A+T−N−	6	69.50 ± 10.60	4|2 (66.67%)	10.50 ± 4.32	2.17 ± 1.94	0.43 ± 0.13	0.07 ± 0.03	25.20 ± 3.54	0.75 ± 0.61	9.33 ± 1.86
A+T+N−	7	70.40 ± 5.75	6|1 (85.71%)	11.30 ± 5.12	3.36 ± 2.06	1.42 ± 0.70	0.27 ± 0.13	26.30 ± 4.03	0.36 ± 0.24	9.21 ± 1.35
A−T+N−	3	77.97 ± 2.38	3|0 (100.00%)	5.33 ± 0.58	4.00 ± 1.00	0.78 ± 0.15	0.12 ± 0.04	21.70 ± 5.86	0.67 ± 0.29	8.00 ± 1.00
aMCI	48	71.50 ± 6.67^f^, ^g^	27|21 (56.25%)	10.80 ± 4.13	3.14 ± 2.12	0.93 ± 0.68^f^, ^h^	0.16 ± 0.12^f^, ^h^	26.0 ± 3.16^f^, ^h^, ^i^	0.37 ± 0.22	9.36 ± 1.18^h^, ^i^
naMCI	21	72.00 ± 7.32^f^, ^g^	8|13 (38.10%)	9.48 ± 4.24	2.18 ± 1.53	0.80 ± 0.83^f^, ^h^	0.13 ± 0.18^f^, ^h^	26.9 ± 2.67^f^, ^h^, ^i^	0.47 ± 0.11	9.10 ± 0.77^h^
mdMCI	67	70.90 ± 7.32^f^, ^g^	35|32 (52.24%)	10.40 ± 4.23	2.75 ± 2.86	0.87 ± 0.77^f^, ^h^	0.14 ± 0.12^f^, ^h^	26.6 ± 3.30^f^, ^h^, ^i^	0.39 ± 0.20	8.85 ± 1.34^h^
AD	64	73.90 ± 5.51^f^, ^g^	38|26 (59.38%)	10.50 ± 4.45	3.10 ± 2.17	1.51 ± 0.86	0.25 ± 0.16	19.00 ± 5.46	1.24 ± 0.82^j^, ^k^, ^l^	8.00 ± 1.51
EOAD	29	59.90 ± 6.68	19|10 (65.52%)	11.50 ± 4.12	3.09 ± 2.00	1.74 ± 0.95	0.28 ± 0.18	18.10 ± 5.33	1.09 ± 0.68^j^, ^k^, ^l^	8.47 ± 1.90
AD + VaD	17	74.60 ± 4.17^f^, ^g^	10|7 (58.82%)	9.12 ± 5.26	2.65 ± 1.99	1.45 ± 0.87	0.25 ± 0.17	19.60 ± 4.33	1.03 ± 0.42^j^, ^k^, ^l^	8.09 ± 1.31
bvFTD	29	66.10 ± 7.48^f^	11|18 (37.93%)	10.70 ± 4.64	2.76 ± 1.90	0.40 ± 0.23^f^, ^h^, ^i^	0.05 ± 0.03^f^, ^h^, ^i^, ^j^	23.60 ± 4.93^f^, ^h^	1.04 ± 0.69^j^, ^k^, ^l^	9.54 ± 1.13^h^, ^i^
LBD	19	75.40 ± 5.97^f^, ^g^	12|7 (63.16%)	8.84 ± 3.27	2.40 ± 1.66	0.77 ± 0.43^f^, ^h^	0.12 ± 0.08^f^, ^h^	18.50 ± 6.32^j^, ^k^, ^l^	1.12 ± 0.45^j^, ^k^, ^l^	6.97 ± 1.05^f^, ^j^, ^k^, ^l^

*Note*: Values are *n* (%) or mean ± SD. Only groups with*n* > 15 were included in the statistical analyses. *P*‐values refer to analysis of variance models, followed by post hoc pairwise comparisons (Bonferroni‐corrected for multiple comparisons, R Software), or chi‐square test. Significance was considered for *p*‐values < 0.05.

Abbreviations: AD, dementia due to Alzheimer disease; aMCI, amnesic MCI; bvFTD, behavioral frontotemporal dementia; CDR, Clinical Dementia Rating scale; CSF, cerebrospinal fluid; EEG, electroencephalographic; EOAD, early onset AD; F, female; IAF, individual alpha frequency; LBD, Lewy body dementia; M, male; MCI, mild cognitive impairment; mdMCI, multidomain MCI; MMSE, Mini‐Mental State Examination; naMCI, nonamnesic MCI; VaD, vascular dementia.

^a^ Versus A+.

^b^ Versus A+T+N+.

^c^ Versus A+T−N+.

^d^ Versus A−T−N−.

^e^ Versus A−T−N+.

^f^ Versus EOAD.

^g^ Versus bvFTD.

^h^ Versus AD.

^i^ Versus mixed AD and VaD.

^j^ Versus aMCI.

^k^ Versus naMCI.

^l^ Versus mdMCI.

**FIGURE 1:**
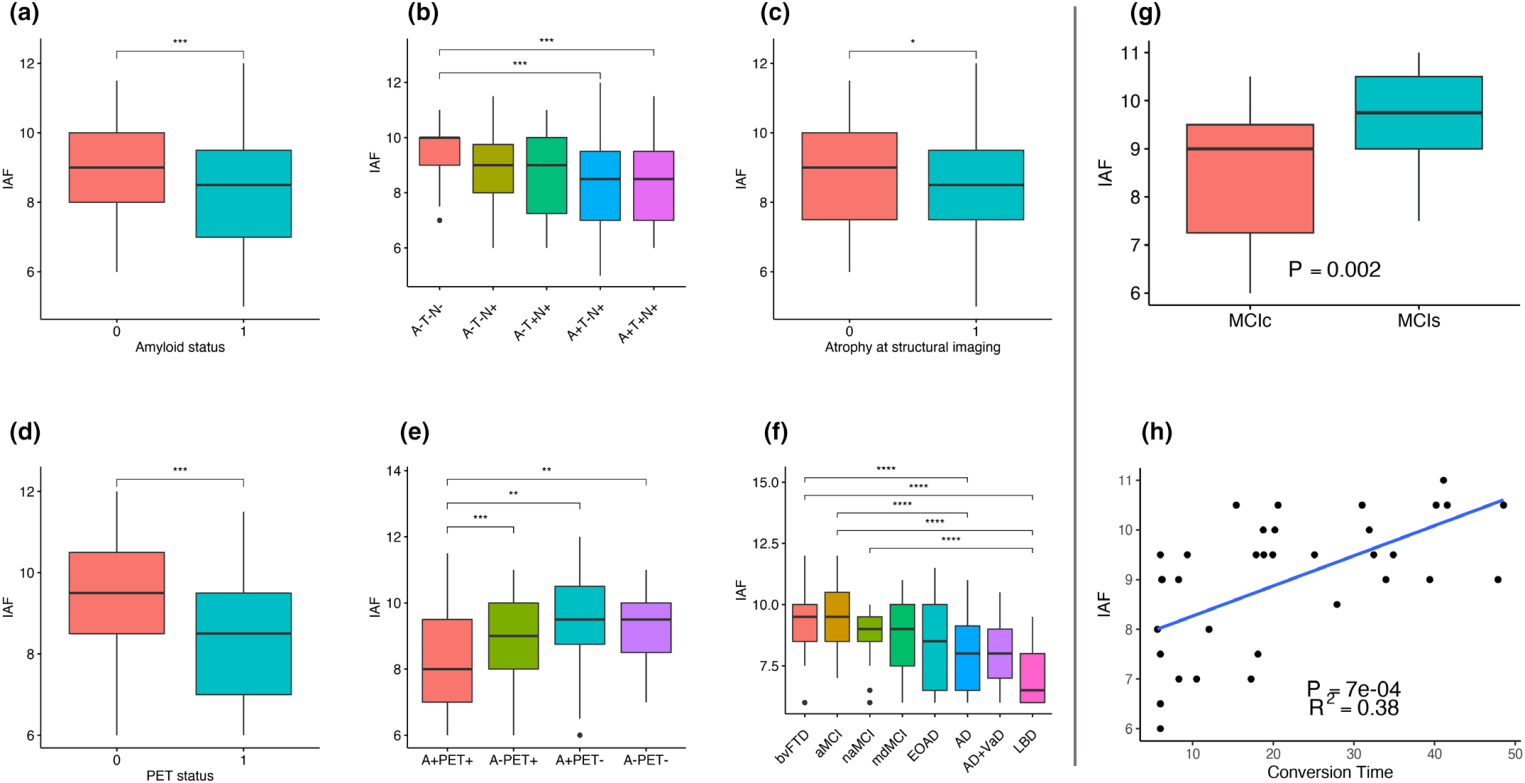
(a–g) Boxplots of individual alpha frequency (IAF) in patients stratified according to: (a) amyloid status, (b) AT(N) groups, (c) evidence of atrophy on structural neuroimaging, (d) positron emission tomography (PET) status, (e) a combination of amyloid and PET status, (f) clinical syndromes, and (g) converter status in A+ mild cognitive impairment (MCI) patients. *P*‐values refer to age‐/sex‐/education‐adjusted analysis of variance models, followed by post hoc pairwise comparisons (Bonferroni‐corrected for multiple comparisons, *p < 0.05, **p < 0.01, ***p < 0.001, ****p < 0.0001, R Software). (h) Linear regression model showing that baseline IAF (Hz) in A+ MCI patients predicted time of conversion (months) to full‐blown Alzheimer disease (AD) dementia, adjusting for age, sex, and education (*p* < 0.05, R software). The *R* ^2^ goodness of fit statistic evaluated the model's performance. aMCI, amnesic MCI; bvFTD, behavioral frontotemporal dementia; EOAD, early onset AD; LBD, Lewy body dementia; MCIc, imminent MCI converters; MCIs, stable MCI; mdMCI, multidomain MCI; naMCI, nonamnesic MCI; VaD, vascular dementia.

Demographic and clinical features of AT(N) groups are reported in Table 1. IAF was significantly higher in A– than in A+ subjects and in A–T–N– than in the A+T+N+ and A+T–N+ groups (Figure 1a,b). ROC analysis confirmed the discriminatory ability of IAF in distinguishing A–T–N– from A+T+N+ and A+T–N+ subjects (area under the curve [AUC] = 0.71, 95% confidence interval [CI] = 0.57–0.85). When considering single biomarkers of neurodegeneration, IAF was significantly higher in PET– (*n* = 44) than PET+ (*n* = 244) subjects (*p* < 0.01), and in Atrophy– (*n* = 144) than in Atrophy+ (*n* = 231) patients (*p* = 0.04; Figure 1c,d). After combining amyloid and FDG‐PET status, A+/PET+ patients showed significantly lower IAF values than A–/PET+ (*p* < 0.01), A+/PET– (*p* = 0.02), and A–/PET– subjects (*p* = 0.01; Figure 1e).

Demographic and clinical features of clinical diagnosis groups are reported in Table 1. IAF was significantly lower in AD dementia patients than in MCI groups (AUC = 0.69, 95% CI = 0.54–0.84) and bvFTD subjects (AUC = 0.75, 95% CI = 0.65–0.85), in mixed dementia subjects than in aMCI (AUC = 0.75, 95% CI = 0.61–0.89) and bvFTD patients (AUC = 0.77, 95% CI = 0.62–0.91), and in LBD than in MCI (AUC = 0.84, 95% CI = 0.68–0.99) and bvFTD patients (AUC = 0.87, 95% CI = 0.76–0.99; Figure 1f).

IAF was significantly lower in A+ MCIc (*n* = 23, mean age = 72.90 ± 6.12 years) than in A+ MCIs (*n* = 19, mean age = 70.30 ± 7.20 years) patients (AUC = 0.66, 95% CI = 0.51–0.82; Figure 1g). IAF also predicted time of conversion in A+ MCI patients who transitioned to AD dementia (*p* < 0.001, *R* ^2^ = 0.38; Figure 1h).

## DISCUSSION

In this cross‐sectional and longitudinal retrospective study, we investigated the potential of IAF as an accessible EEG‐derived parameter to enhance dementia differential diagnosis and prognosis. As the global burden of dementia rises, there is an increasing need for cost‐effective and minimally invasive diagnostic tools, especially with advancing therapeutic interventions.

Correlation analysis revealed that IAF may capture distinct neurophysiological aspects independent from age, disease duration, and pTau/Aβ42. IAF showed instead a small correlation with degree of cognitive impairment, as previously observed [13].

Within the AT(N) classification system [5], IAF exhibited distinct variations across different AT(N) groups. Specifically, IAF was lower in A+ subjects compared to A– counterparts, and in A+T+N+ and A+T–N+ groups compared to A–T–N– subjects (Figure 1). The ROC analysis provided insight into the moderate ability of IAF to distinguish between different ATN groups. Examining IAF in relation to imaging neurodegeneration biomarkers, FDG‐PET, and structural neuroimaging, N+ patients consistently had lower IAF values than N– subjects (Figure 1). These findings align with previous literature, confirming the association of disrupted alpha rhythms with hypometabolism on FDG‐PET and atrophy on MRI [14]. Notably, when integrating A status with FDG‐PET findings, A+/PET+ individuals demonstrated lower IAF values compared to other subgroup, suggesting that lower IAF values may align with poorer outcomes in cognitively impaired patients [15].

Beyond the AT(N) system, IAF showed moderate to good discriminatory capabilities among specific dementia clinical subtypes, particularly highlighting the role of IAF in distinguishing between later stages of dementia and in reflecting progression, rather than serving as a tool for distinguishing early stage clinical phenotypes. Although not all subjects’ psychotropic medication data were available, AD and LBD patients showed the lowest IAF values, aligning with studies proposing cholinergic failure as the basis for progressive EEG slowing in AD continuum and LBD [1, 2, 13].

Furthermore, our study ventured into predicting disease progression, particularly in the context of MCI conversion to AD dementia. Aligning partially with previous evidence [4], IAF discriminated imminent MCI converters to AD dementia. This addresses a critical need for early prognostic biomarkers and timely intervention, particularly in light of future disease‐modifying therapies for AD.

In conclusion, IAF emerges as a valuable asset in dementia differential diagnosis and prognosis. Its accessibility, coupled with its associations with amyloid status and neurodegeneration, underscores its potential to aid the diagnostic landscape. Furthermore, its prognostic value in identifying A+ MCI patients at imminent risk of conversion to dementia positions IAF as a critical tool in the pursuit of timely and targeted interventions, particularly in the era of emerging disease‐modifying therapies for AD.

## AUTHOR CONTRIBUTIONS

**Giordano Cecchetti:** Conceptualization; investigation; formal analysis; writing – original draft; writing – review and editing. **Federica Agosta:** Conceptualization; investigation; formal analysis; writing – original draft; writing – review and editing. **Elisa Canu:** Investigation; formal analysis; writing – original draft; writing – review and editing. **Silvia Basaia:** Investigation; formal analysis; writing – original draft; writing – review and editing. **Giulia Rugarli:** Investigation; formal analysis; writing – review and editing. **Davide G. Curti:** Investigation; formal analysis; writing – review and editing. **Federico Coraglia:** Investigation; formal analysis; writing – review and editing. **Marco Cursi:** Investigation; formal analysis; writing – review and editing. **Edoardo G. Spinelli:** Investigation; formal analysis; writing – review and editing. **Roberto Santangelo:** Investigation; formal analysis; writing – review and editing. **Francesca Caso:** Investigation; writing – review and editing. **Giovanna Franca Fanelli:** Investigation; writing – review and editing. **Giuseppe Magnani:** Investigation; writing – review and editing. **Massimo Filippi:** Conceptualization; supervision; writing – review and editing.

## CONFLICT OF INTEREST STATEMENT

G.C. has received speaker honoraria from Neopharmed Gentili. F.A. is an associate editor of *NeuroImage: Clinical*, has received speaker honoraria from Biogen Idec, Italfarmaco, Roche, Zambon, and Eli Lilly, and receives or has received research support from the Italian Ministry of Health, the Italian Ministry of University and Research, AriSLA (Fondazione Italiana di Ricerca per la SLA), the European Research Council, the EU Joint Programme–Neurodegenerative Disease Research, and Foundation Research on Alzheimer Disease (France). E.C. receives research support from the Italian Ministry of Health. S.B. receives research support from the Italian Ministry of Health. E.G.S. receives research support from the Italian Ministry of Health. M.F. is editor‐in‐chief of *Journal of Neurology* and an associate editor of *Human Brain Mapping*, *Neurological Sciences*, and *Radiology*. He has received compensation for consulting services from Alexion, Almirall, Biogen, Merck, Novartis, Roche, and Sanofi; for speaking activities from Bayer, Biogen, Celgene, Chiesi Italia, Eli Lilly, Genzyme, Janssen, Merck‐Serono, Neopharmed Gentili, Novartis, Novo Nordisk, Roche, Sanofi, Takeda, and Teva; for participation on advisory boards from Alexion, Biogen, Bristol Myers Squibb, Merck, Novartis, Roche, Sanofi, Sanofi‐Aventis, Sanofi‐Genzyme, and Takeda; and for scientific direction of educational events from Biogen, Merck, Roche, Celgene, Bristol Myers Squibb, Lilly, Novartis, and Sanofi‐Genzyme. He receives research support from Biogen Idec, Merck‐Serono, Novartis, Roche, the Italian Ministry of Health, the Italian Ministry of University and Research, and Fondazione Italiana Sclerosi Multipla. None of the other authors has any conflict of interest to disclose.

## Data Availability

The dataset and codes used for this study will be made available by the corresponding author on request. The data are not publicly available due to privacy or ethical restrictions.
